# The public health emergency management system in China: trends from 2002 to 2012

**DOI:** 10.1186/s12889-018-5284-1

**Published:** 2018-04-11

**Authors:** Mei Sun, Ningze Xu, Chengyue Li, Dan Wu, Jiatong Zou, Ying Wang, Li Luo, Mingzhu Yu, Yu Zhang, Hua Wang, Peiwu Shi, Zheng Chen, Jian Wang, Yueliang Lu, Qi Li, Xinhua Wang, Zhenqiang Bi, Ming Fan, Liping Fu, Jingjin Yu, Mo Hao

**Affiliations:** 1 Research Institute of Health Development Strategies & Collaborative Innovation Center of Social Risks Governance in Health, Fudan University, 177 box, 130 Dong’an Road, Shanghai, 200032 China; 2 Key Lab of Health Technology Assessment, National Health Commission of the People’s Republic of China (Fudan University), School of Public Health, Fudan University, Shanghai, China; 3 Shanghai Health Education Institution, Shanghai, China; 4 Bureau of Disease Control and Prevention of National Health Commission of the People’s Republic of China, Beijing, China; 5 Health and Family Planning Commission of Hubei Province, Wuhan, China; 6 Health and Family Planning Commission of Jiangsu Province, Nanjing, China; 7 Zhejiang Academy of Medical Sciences, Hangzhou, China; 8 National Grassroots Health Prevention Group, Shanghai, China; 9 Chinese Center for Disease Control and Prevention, Beijing, China; 10 Jiangsu Provincial Center for Disease Control and Prevention, Nanjing, China; 11 Hebei Provincial Center for Disease Control and Prevention, Shijiazhuang, China; 12 Gansu Provincial Center for Disease Control and Prevention, Lanzhou, China; 13 Shandong Provincial Center for Disease Control and Prevention, Jinan, China; 14 Jilin Provincial Center for Disease Control and Prevention, Changchun, China; 15 Xinjiang Provincial Center for Disease Control and Prevention, Urumqi, China

**Keywords:** Public health emergency management system, China, Trend, Preparation, Readiness, Response, Recovery

## Abstract

**Background:**

Public health emergencies have challenged the public health emergency management systems (PHEMSs) of many countries critically and frequently since this century. As the world’s most populated country and the second biggest economy in the world, China used to have a fragile PHEMS; however, the government took forceful actions to build PHEMS after the 2003 SARS outbreak. After more than one decade’s efforts, we tried to assess the improvements and problems of China’s PHEMS between 2002 and 2012.

**Methods:**

We conducted two rounds of national surveys and collected the data of the year 2002 and 2012, including all 32 provincial, 139 municipal, and 489 county CDCs. The municipal and county CDCs were selected by systematic random sampling. Twenty-one indicators of four stages (preparation, readiness, response and recovery) from the National Assessment Criteria for CDC Performance were chosen to assess the ten-year trends.

**Results:**

At the preparation stage, organization, mechanisms, workforce, and stockpile across all levels and regions were significantly improved after one decade’s efforts. At the readiness stage, the capability for formulating an emergency plan was also significantly improved during the same period. At the response stage, internet-based direct reporting was 98.8%, and coping scores were nearly full points of ten in 2012. At the recovery stage, the capabilities were generally lower than expected.

**Conclusions:**

Due to forceful leadership, sounder regulations, and intensive resources, China’s PHEMS has been improved at the preparation, readiness, and response stages; however, the recovery stage was still weak and could not meet the requirements of crisis management and preventive governance. In addition, CDCs in the Western region and counties lagged behind in performance on most indicators. Future priorities should include developing the recovery stage, establishing a closed feedback loop, and strengthening the capabilities of CDCs in Western region and counties.

## Background

Since the early twenty-first century, frequently appearing public health emergencies such as severe acute respiratory syndrome (SARS), Middle Eastern respiratory syndrome, and Ebola have threatened population health and social stability [1]. This has critically challenged the public health emergency management systems (PHEMSs) of many countries [2], especially developing countries. The global community quickly reached a consensus on the development of the PHEMSs [3]. In 2005, the 58th World Health Assembly (WHA) adopted the revised International Health Regulations, which instructed the World Health Organization (WHO) member states to collaboratively confront public health emergencies of global concern. A World Health Report in 2007 also focused on global public health security in the twenty-first century. The Ebola outbreak in 2014–2015 has pushed the process of WHO reform into high gear [4], giving top priority to changes in the WHO’s emergency operations and a need to build resilient health systems that can withstand epidemics.

China has the largest population and the second biggest economy in the world. China has played an increasingly important role in preventing and controlling the global spread of epidemics in recent years and gradually changed from aid recipient to aid donor [5]. China used to have a fragile PHEMS; however, the 2003 SARS outbreak exposed many weaknesses and problems [6], such as an ineffective response system, lagging epidemiological field investigation and laboratory testing skills, and inaccurate and untimely information communication. These aroused the public’s horror and international community’s blame. The central government urged governments at different levels to make political commitments and take forceful actions to build the PHEMS.

After more than one decade’s efforts, what are the trends of China’s PHEMS? What are the improvements and remaining problems? What are the implications for China and global health security? In recent years, the development of PHEMS has received increased attention in the literatures. Some researchers expressed the importance of PHEMS and the progress after SARS qualitatively [7, 8]. Others quantitatively accessed the trends using regional data, usually at a certain level or within a certain province or city [9–12]. Time spans were restricted to early-phase usually around 2005 [13]. To our knowledge, little evidence could tell the differences that happened in China’s PHEMS in this decade.

Based on two national surveys in 2006 and 2013, we previously reported that resource allocation of CDCs increased and the general completeness of PHEMS improved between 2002 and 2012 [14]. However, what measures PHEMS carried out and how it changed still remained unclear. This paper will attempt to answer these questions specifically.

This article consists of the follows. The next section provides details on methodology,including sampling, indicator selection and measurements, data collection, and data analysis methods. The third section shows the results, followed by discussion corresponding to the results. The final section is about conclusion and policy implications.

## Methods

### Sample

The survey methods have previously been published [14]. Briefly, we conducted two rounds of cross-sectional surveys in 2006 and 2013. The two surveys were retrospective and selected the same agencies in the two rounds. The survey of 2006 collected the data from 2002 to 2005, and the survey of 2013 collected data of 2012. We conducted a multistage sampling to select CDCs at different administration levels, selected all 32 provincial CDCs and used systematic random sampling to select municipal and county CDCs. As governmental funding is the most critical control point of public health emergency management for the CDCs [15],we used “governmental funding to CDCs per thousand people” as a basis to determine sample size [16]. A sample size of 123 municipal and 457 county CDCs was calculated based on the following formula [17].

$$ n={\left[\frac{\left({u}_{\alpha }+{u}_{\beta}\right)\times \sigma }{\delta}\right]}^2 $$

where n is the number of the minimal sample size; αis the probability of type I error, and β is the probability of type II error, here α = 0.05,β = 0.05; *u*_α_and *u*_β_are standard normal distribution values corresponding to α and β respectively;σis the population standard deviation, hereσ = 404.3 yuan; δ is the allowable error. For municipal CDCs, δ = 54.9yuan, σ = 210.0 yuan. For county-level CDCs, δ = 62.5yuan, σ = 404.3yuan (1 U.S. dollar = 6.6 yuan).

The municipal and county level CDCs were all selected through random sampling. The sampling process was conducted based on the national standard coding (GB coding, the corresponding administrative regional code which is unique for each city or county [14]). We used a computer-generated random number to identify the first institution, and then selected every third municipal CDC and every sixth county level CDC. Finally, we selected 32 provincial CDCs, 139 municipal CDCs, and 489 county CDCs.

The study was approved by the former Ministry of Health (MOH) in China and reviewed by the Medical Research Ethics Committee at the School of Public Health of Fudan University.

### Measures

We selected twenty-one indicators associated with the PHEMS from the *National Assessment Criteria for CDC Performance*. Based on the crisis management theory which was commonly used in the field of public emergency management [18, 19], the whole process was divided into four stages including preparation, readiness, response and recovery [20]. According to the framework, we grouped the indicators into 4 stages and 13 capabilities. Table 1 showed the features, units and measurements of these indicators.

**Table 1:** Measurements of public health emergency management system

Stage	Capability	Indicator	Unit	Response measurement and indicator calculation
1.Preparation	1.1Organization	Percentage of establishing emergency response office	%	yes/no; number of CDCs’ responses/sample size
		Percentage of forming leadership group	%	
		Percentage of forming expert panel	%	
	1.2Mechanisms	Percentage of building information sharing mechanism	%	
		Percentage of building on-site treatment mechanism	%	
		Percentage of building material deployment mechanism	%	
	1.3Workforce	Average number of emergency response personnel	Person	number; total number of personnel/sample size
	1.4Stockpile	Percentage of fully stockpiling emergency resources	%	yes/no; number of stockpiling emergency resources/fully stockpiling emergency resources
2.Readiness	2.1Planning	Percentage of formulating emergency response plan	%	yes/no; number of CDCs’ responses/sample size
	2.2Training	Average length of emergency response training	Day/ person	total days of emergency response training/total emergency response personnel
	2.3Exercising	Average times of exercises of emergency response plan	Number of times	total times of exercises /sample size
	2.4Monitoring	Disease surveillance and analytical period	Frequency	by day, week, ten days, month, quarter, year
	2.5Direct report	Percentage of internet direct report building	%	number; number of internet direct reports/total reports
3.Response	3.1Reporting	Percentage of timely reporting	%	number; number of timely reports/total reports
	3.2Coping	Confirmation Score	Points	Ten-point scale, full points of 10 = good; Total scores/sample size
		Specific Preparedness Score	Points	
		On-scene/field handling/disposal score	Points	
		Implementation score for control measures	Points	
4.Recovery	4.1Archiving	Archive of relevant materials	Points	
	4.2Analyzing	Analytical report and impact evaluation	Points	
	4.3Concluding	Concluding report	Points	

Note *CDC* means Center for Disease Prevention and Control

According to the *National Regulations on Public Health Emergency Management* [21], each sampled CDC graded five public health emergencies handled in the year before the survey with the full mark of 10 points for each indicator; at CDCs where the total numbers of handled public health emergencies were fewer than five, all public health emergencies were graded instead.

### Quality control

The Bureau of Disease Prevention and Control of the former MOH approved and organized two rounds of field surveys, and 32 provincial Health Departments coordinated data collection.

A pilot survey was conducted to ensure validity and reliability. After receiving uniform training from the MOH, the provincial quality supervisors trained investigators from sampled CDCs in their corresponding provinces. The investigators collected relevant data from sampled CDCs and submitted the completed questionnaires to their provincial quality supervisors via e-mail or CD-ROM. Simultaneously, paper copies with official stamps were submitted.

The second round of survey data were obtained from National Disease Control and Prevention Performance Evaluation Platform. The quality control process was set up and carried out by the platform with backend logic judgments and audit procedures.

As the final step of quality control in both surveys, research group rechecked data and contacted CDCs with abnormal or absent values via email or phone. Finally, the overall response rate was 95.8% in 2002 and 99.5% in 2012.

### Data analysis

We established a dataset using Excel 2013(Microsoft Redmond WA). We only used the data of the year 2002 and 2012 for analysis. After data cleaning and sorting, descriptive analysis and statistical tests were performed using SPSS 21.0 (IBM SPSS, Chicago, IL, USA). We used McNemar’s test to test differences in proportions and paired sample *t* test to test differences in means between 2002 and 2012. Since noticeable differences existed between China’s regions, the division of regions was based on the 2003 *Chinese Economics Yearbook* and the *First National Economic Census*.

## Results

### Preparation stage

Establishing organization comprised building an emergency response office and forming a leadership group and an expert panel. The average percentage of CDCs with an emergency response office was 61.6% in 2002 and 95.0% in 2012. The average percentages with a leadership group and an expert panel were 47.9% and 78.6% in 2002 and 95.7% and 96.8% in 2012, respectively. Similar trends also occurred across different levels and regions (Table 2).

**Table 2:** Evaluation of preparation and readiness stage by levels and regions: 2002 and 2012 (differences in proportions)

Indicators	2002		2012		Growth (%)	*p*-value
	n	%	n	%		
1.1 Organization						
% of establishing emergency response office	632	61.6	644	95.0	54.2	0.5110
Provincial	29	64.3	31	96.8	50.5	0.0310
Municipal	135	56.3	138	96.4	71.2	0.0080
County	468	51.1	475	94.5	84.9	0.1560
East	124	55.6	129	93.0	67.3	0.1040
Central	254	54.7	255	97.6	78.4	0.6910
West	254	49.4	260	93.5	89.3	0.5860
% of forming leadership group	632	47.9	644	95.7	99.8	< 0.0001
Provincial	29	78.6	31	96.8	23.2	0.0210
Municipal	135	47.4	138	97.1	104.9	< 0.0001
County	468	46.2	475	95.2	106.1	< 0.0001
East	124	53.2	129	93.8	76.3	< 0.0001
Central	254	50.0	255	97.3	94.6	< 0.0001
West	254	43.1	260	95.0	120.4	< 0.0001
% of forming expert panel	632	78.6	644	96.8	23.2	< 0.0001
Provincial	29	82.1	31	93.5	13.9	0.1090
Municipal	135	38.5	138	96.4	150.4	< 0.0001
County	468	30.6	475	84.0	174.5	< 0.0001
East	124	37.9	129	89.1	135.1	< 0.0001
Central	254	39.0	255	92.2	136.4	< 0.0001
West	254	28.5	260	81.2	184.9	< 0.0001
1.2 Mechanism						< 0.0001
% of building information sharing mechanism	632	48.0	644	92.9	93.5	< 0.0001
Provincial	29	67.9	31	93.5	37.7	0.0060
Municipal	135	48.9	138	96.4	97.1	< 0.0001
County	468	46.6	475	91.8	97.0	< 0.0001
East	124	52.4	129	92.2	76.0	< 0.0001
Central	254	46.9	255	96.1	104.9	< 0.0001
West	254	47.0	260	90.0	91.5	< 0.0001
% of building on-site treatment mechanism	632	49.1	644	93.0	89.4	< 0.0001
Provincial	29	79.3	31	93.5	17.9	0.1090
Municipal	135	48.1	138	95.7	99.0	< 0.0001
County	468	47.4	475	92.2	94.5	< 0.0001
East	124	54.8	129	91.5	67.0	< 0.0001
Central	254	46.9	255	95.7	104.1	< 0.0001
West	254	48.4	260	91.2	88.4	< 0.0001
% of building response material deployment mechanism	632	39.6	644	90.1	127.5	< 0.0001
Provincial	29	67.9	31	90.3	33.0	0.0350
Municipal	135	39.3	138	95.7	143.5	< 0.0001
County	468	38.0	475	88.4	132.6	< 0.0001
East	124	45.2	129	91.5	102.4	< 0.0001
Central	254	40.2	255	93.3	132.1	< 0.0001
West	254	36.4	260	86.2	136.8	< 0.0001
2.1 Emergency plan						
% of making emergency plans	632	40.6	644	89.9	121.4	< 0.0001
Provincial	29	42.9	31	93.5	117.9	< 0.0001
Municipal	135	38.5	138	89.1	131.4	< 0.0001
County	468	41.0	475	89.9	119.3	< 0.0001
East	124	35.5	129	86.0	142.3	< 0.0001
Central	254	46.1	255	92.5	100.7	< 0.0001
West	254	37.5	260	89.2	137.9	< 0.0001
2.4 Disease surveillance frequency	560	–	614	–	–	–
Per day	16	2.9	29	4.7	62.1	0.0400
Per week	14	2.5	141	23.0	820.0	< 0.0001
Per ten days	71	12.7	10	1.6	−87.4	< 0.0001
Per month	324	58.0	391	63.7	9.8	< 0.0001
Per quarter	71	12.7	26	4.2	−66.9	< 0.0001
Per year	63	11.3	17	2.8	−75.2	< 0.0001

The capability for building mechanisms in terms of information sharing and on-site treatment increased by 93.5% and 89.4%, respectively. Increasing by 127.5%, response-material deployment mechanism gained the highest growth rate. Municipal CDCs had the highest percentages, followed by provincial and county CDCs. The central region not only had the highest percentages, but also experienced the highest growth rate.

Average number of emergency response personnel per CDC increased from 15 in 2002 to 31 in 2012, which was significant. In 2012, provincial CDCs had the highest number of personnel (*n* = 92), followed by municipal (*n* = 47) and county (*n* = 22) CDCs. Moreover, the average number decreased from eastern (*n* = 35) to western regions (*n* = 29) (Table 3).

**Table 3:** Evaluation of preparation and readiness stage by levels and regions: 2002 and 2012 (differences in means)

Indicators	2002		2012		Growth (%)	*p*-value
	n	Mean	n	Mean		
1.3 Personnel	475	15	623	31	106.7	< 0.0001
Provincial	26	28	30	92	228.6	< 0.0001
Municipal	102	22	134	47	113.6	< 0.0001
County	347	12	459	22	83.3	< 0.0001
East	124	14	125	35	150	< 0.0001
Central	254	15	252	31	106.7	< 0.0001
West	254	16	246	29	81.3	< 0.0001
1.4 Emergency stockpile	632	16.7	601	41.2	146.7	< 0.0001
Provincial	29	36.7	30	74.2	102.2	< 0.0001
Municipal	135	20.7	127	56.8	174.4	< 0.0001
County	468	14.3	444	34.5	141.3	< 0.0001
East	124	22.7	121	56.7	149.8	< 0.0001
Central	254	18.2	249	42.5	133.5	< 0.0001
West	254	12.2	231	31.7	159.8	< 0.0001
2.2 Length of response training	415	9.7	620	14.6	50.5	0.6060
Provincial	20	25.0	30	44.3	77.2	0.0060
Municipal	84	8.7	132	21.1	142.5	0.1600
County	311	9.0	458	10.8	20.0	0.3290
East	111	7.1	123	14.8	108.5	0.3360
Central	155	11.8	253	15.3	29.7	0.0010
West	149	9.2	244	13.9	51.1	0.1770
2.3 Times of Emergency exercise	318	2.3	619	2.2	−4.3	< 0.0001
Provincial	16	1.1	30	1.5	36.4	< 0.0001
Municipal	63	2.1	133	1.7	−19.0	< 0.0001
County	239	2.5	456	2.4	−4.0	< 0.0001
East	107	1.4	124	1.8	28.6	0.0090
Central	112	2.9	252	2.1	−27.6	< 0.0001
West	99	2.9	243	2.7	−6.9	0.0200

The percentage of fully stockpiling emergency resources significantly increased from 16.7% in 2002 to 41.2% in 2012. Provincial CDCs had the highest percentage (74.2%) in 2012 and increased by 102.2%, whereas county CDCs had the lowest percentage (34.5%) in 2012 and increased by 141.3%. Nevertheless, the average percentage at each administrative level did not meet the corresponding performance assessment criteria. Average percentages of fully stockpiling emergency resources decreased from eastern (56.7%) to western (31.7%) regions.

### Readiness stage

The mean percentage of formulating emergency plan increased from 40.6% in 2002 to 89.9% in 2012, statistically significantly increasing by 121.4%. Provincial CDCs had the highest percentage (93.5%) in 2012, and the difference between municipal (89.1%) and county CDCs (89.9%) was not significant. CDCs in central region had the highest percentage (92.5%), followed by western (89.2%) and eastern (86.0%) regions (Table 2).

The average length of emergency response training increased from 9.7 days per person in 2002 to 14.6 days per person in 2012; however, this 50.5% increase was not statistically significant. Provincial CDCs had the highest average length of response training (44.3 days per person), followed by municipal and county CDCs (Table 3).

Comparing the statistics in 2002 and 2012, the average times of exercises did not change with statistical significance. In 2012, county CDCs had higher average times of exercises than did municipal (1.7) and provincial (1.5) CDCs; nevertheless, only provincial CDCs had increased average times of exercises during the past decade. From regional perspective, the average times of exercises decreased from western (2.7) to eastern (1.8) regions (Table 3).

There were 63.7% and 23.0% of disease surveillances conducted per month and per week in 2012, respectively. Compared with statistics in 2002, frequencies of daily, weekly, and monthly surveillance analysis increased, among which weekly surveillance analysis increased with statistical significance. Meanwhile, the frequencies of disease surveillance analysis per ten days, quarter, and year decreased with statistical significance (Table 2).

### Response stage

According to “contingency rules of paroxysmal public health events”, public health emergency events are classified into four levels (I, II, III and IV), with severity decreasing from Level I to Level IV. In 2012, there were 3092 public health emergencies directly reported via the Disease Surveillance Information Management System, which accounted for 98.8%.The percentage of timely reporting by county CDCs emergency levels in 2012 was presented in Table 4. Moreover, the average scores for indicators of coping capability were high in 2012 (Table 4).

**Table 4:** Percentage of timely reporting by county CDCs by emergency levels in 2012

Region	Level I	Level II	Level III	Level IV	Unclassified	Total
East	100.0	-	100.0	57.4	59.4	59.5
Central	-	-	100.0	92.9	96.4	96.3
West	75.0	100.0	92.3	91.5	89.0	89.7
Total	83.3	100.0	94.1	78.7	84.1	83.6

Note “-” means there was no such emergency at the corresponding level. The severity of public health emergency decreased from level I to level IV. *CDC means* Center for Disease Prevention and Control

### Recovery stage

The average scores for capabilities at recovery stage were lower than those for capabilities at response stage. The average score for data archiving was 8.33, then followed by those for data analyzing (5.83) and concluding (5.69) (Table 5).

**Table 5:** Evaluation of coping capability and recovery stage by levels and regions in 2012

Level/region	n	Emergency confirmation		Response preparedness		On-site response		Implementation of control measures		Archiving		Analyzing		Concluding	
		Points	95% CI	Points	95% CI	Points	95% CI	Points	95% CI	Points	95% CI	Points	95% CI	Points	95% CI
Average	271	9.61	9.52–9.69	9.25	9.15–9.34	9.21	9.12–9.30	9.17	9.08–9.26	8.33	8.15–8.52	5.83	5.59–6.07	5.69	5.45–5.95
Provincial	25	9.73	9.53–9.88	9.75	9.66–9.83	9.77	9.71–9.83	9.65	9.54–9.76	7.98	7.46–8.48	5.85	5.18–6.49	6.17	5.57–6.80
Municipal	102	9.85	9.78–9.92	9.44	9.33–9.53	9.43	9.35–9.51	9.46	9.38–9.54	8.54	8.27–8.81	5.37	4.99–5.76	5.34	4.96–5.70
County	114	9.27	9.08–9.46	8.82	8.63–9.02	8.73	8.54–8.93	8.63	8.44–8.83	8.22	7.90–8.53	6.40	6.00–6.80	5.93	5.57–6.31
East	70	9.65	9.50–9.80	9.20	9.01–9.36	9.24	9.07–9.40	9.03	8.84–9.20	7.80	7.41–8.18	5.74	5.29–6.20	5.45	5.00–5.94
Central	81	9.54	9.36–9.71	9.23	9.05–9.39	8.98	8.79–9.14	9.09	8.90–9.26	8.73	8.43–9.03	5.44	5.00–5.89	5.38	4.96–5.83
West	120	9.63	9.51–9.74	9.31	9.17–9.43	9.38	9.26–9.49	9.34	9.22–9.46	8.39	8.07–8.68	6.22	5.81–6.60	6.11	5.73–6.46

## Discussion

The main findings indicated that China had made significant progress in the four stages after a decade’s efforts, especially in preparation, readiness, and response stages. This has been demonstrated by other researches [7, 8].

The average percentages of CDCs with an emergency response office, a leadership group and an expert panel were 95.0%, 95.7% and 96.8% in 2012, respectively. This suggests that a PHPM system with better leadership has been established in China. Soon after the SARS outbreak, Chinese governments at different levels were urged to establish a SARS headquarters at CDCs to shoulder the responsibilities of unified leadership and command during public health emergencies. The *Emergency Response Law of the People’s Republic of China* issued in 2007 formally and strongly stipulated the establishment of the emergency management system that urged unified leadership, comprehensive coordination, categorized management, graded responsibility, and territorial management.

The capability for building mechanisms comprised of information sharing, on-site treatment and response-material deployment increased to more than 95% in 2012. Boosted by the SARS outbreak in 2003, various authorities consecutively issued a series of regulations that standardized the PHEMS in terms of macro-level management, professional categories, disposal processes, etc. From the perspective of macro-level management, regulations included emergency management [22], organizational establishment [23], coordination mechanisms [24], etc. From the perspective of professional categories, regulations standardized the responses to nuclear accidents [25], infectious disease outbreaks [26], etc. From the perspective of disposal processes, regulations clearly guided emergency response plans [27], exercising [28], information reporting [29], etc.

Another notable foundation is that the growth of resources including workforce and stockpile was 106.7% and 146.7%, respectively. Since 2003, intensive investments by governments have contributed to the improvements on the following aspects. First, funding for CDCs across different levels changed from balanced allocation to full fiscal funding after 2003. Total income governmental funding increased from 40.75% in 2002 to 63.3% in 2012 [30]. Second, CDCs’ staff were overall more educated. The percentage of staff with bachelor degree or higher increased from 12.7% in 2002 to 29.4% in 2012 [31]. Last, the total value of fixed assets of all CDCs increased from 0.42 billion CN¥ in 2002 to 12.9 billion CN¥ in 2012 [31]. Available research showed that the quantity and quality of emergency staff, governmental-funding level, and fixed assets played important roles in improving the implementation of CDCs’ capabilities in the PHEMS [15].

A firm leadership, a favorable mechanism and sufficient resources are the key elements of a well-developed PHPMS [32]. It is undeniable that the PHEMS’ achievements in the past decade are remarkable. China’s active and constructive contributions have been highly valued by the global community; for example, China’s response to H7N9 in 2013 was recognized as “exemplary” by the WHO [33]. The three leading guarantees of China could be referenced by developing and other underdeveloped countries.

However, to cope with future challenges in global health security, the following aspects require strengthening. **First, preventive governance is necessary.** The recovery stage capabilities were the weakest, which is far from achieving the standard of full recovery including sustainability, resilience after crisis and feedback to preparation-stage. The prediction, communication, and social services during and after emergencies require improvement.

**Second, balanced development at different regions and levels is very important.** County CDCs in the front lines [34] had the weakest capabilities. One possible reason was that the relevant policies including contingency plan, work specifications, and guidelines were not instructive and operable enough for county CDCs [35]. Another reason was an inequitable distribution of personnel in urban and rural areas [36]. Available data showed that compared with county CDCs, a greater number of personnel with degree higher than bachelor worked at provincial and municipal CDCs [37]. Additionally, the governmental funding per staff for county CDCs in 2012 was 0.1557 million CN¥, which was much lower than the funding at municipal and provincial CDCs (0.2593 and 0.5406 million CN¥, respectively) [38]. From the perspective of regional disparity, CDCs in Western region were the weakest. Reasons include that it had the poorest fiscal capacity to fund CDCs; a limited personnel size; and an inadequate stockpile in terms of working budget, timely reserves, and prompt delivery [39].

**Third, the application of new technologies should keep pace with science and technology development.** For example, the disease surveillance systems need to be integrated with the use of standard data formats and allow the public health community to respond more quickly to public health threats [40]. A Stockpile Management and Tracking System could also be designed and used to manage stockpiles across different levels and regions [41].

### Limitations

The available assessment indicators are relatively narrower in comparison with those such as the Capability Assessment for Readiness and the Target Capabilities List of Homeland Security Exercise and Evaluation Program in the United States.

Nearly half the indicators were binary (“yes” or “no”), so the quality of policy implementation and accountability could not be judged.

Although logic judgments and audit procedures were conducted, recall bias may still exist. Despite these limitations, the main contribution of this paper are the findings based on the data from two rounds of national field surveys conducted in 2002 to 2012 in China. We believe that this contribution is theoretically and practically relevant because the lessons China’s government learned from the 2003 SARS outbreak provide an emergency response framework that can be employed by developing countries.

## Conclusions

Since the 2003 SARS outbreak, China has built an effective PHEMS and achieved comprehensive progress and improvements at preparation, readiness, response, and recovery. Nevertheless, lacks of conceptual crisis management and preventive governance, disparities across regions and levels, and insufficient application of new technologies remain. Future priorities should be to develop the recovery stage, establish a closed-feedback loop between recovery and preparation stages, and strengthen capability-building CDCs in Western areas through increasing governmental funding and improving the quality of response personnel. The guarantees of leadership, regulations, and resources provide useful references for other developing countries.

## Abbreviations

CDC: Center for Disease Prevention and Control
MOH: Ministry of Health
PHEMS: Public Health Emergency Management System
SARS: severe acute respiratory syndrome
WHA: World Health Assembly
WHO: World Health Organization
